# Novel Therapeutic Approaches for Colorectal Cancer Treatment

**DOI:** 10.3390/ijms25042228

**Published:** 2024-02-13

**Authors:** Athanasios G. Papavassiliou, Donatella Delle Cave

**Affiliations:** 1 Department of Biological Chemistry, Medical School, National and Kapodistrian University of Athens, 11527 Athens, Greece; papavas@med.uoa.gr; 2 Institute of Genetics and Biophysics ‘Adriano Buzzati-Traverso’, CNR, 80131 Naples, Italy

According to GLOBOCAN 2020 data, colorectal cancer (CRC) represents the third most common malignancy and the second most deadly cancer worldwide [1,2,3]. In a clinical setting, despite advances in diagnosis and surgical procedures, 20% of patients with CRC present with metastasis at the time of diagnosis due to residual tumor cells that have spread to distant organs prior to surgery, affecting the patient’s survival rate [4]. Standard systemic chemotherapy, alternative therapies targeting mechanisms in cancer progression and metastasis, immunotherapy, and combination therapies are the primary strategies for treating CRC [5,6]. Unfortunately, these treatment strategies are expensive and often lack selectivity in targeting cancer cells, leading to severe toxicity in normal tissues and various side effects [7]. The main purpose of this Editorial is to provide a concise and state-of-the-art overview of novel therapeutic approaches for CRC treatment.

Mutations in different signaling pathways contribute to the initiation, progression, and chemoresistance of CRC, and among them, the overactivation of the phosphoinositide 3-kinase (PI3K)/AKT/mechanistic target of rapamycin (mTOR) signaling axis is of pivotal importance for tumorigenicity [8,9,10]. Although there are conflicting data regarding the effectiveness of agents directed against the PI3K axis in CRC, several studies have shown favorable results for these drugs, whether used in primary or metastatic cases. Among them, Pictilisib, a potent small-molecule class I PI3K inhibitor (PI3Ki), has shown promising results in reducing mucinous colorectal adenocarcinoma (MCA) progression; however, its effectiveness as single-agent therapy is limited due to the potential development of drug-induced resistance [11,12,13]. Kuracha et al. demonstrated that, in MCA cells, Pictilisib-induced adaptive resistance is regulated by the forkhead box O (FOXO)-dependent rebound activity of receptor tyrosine kinases (RTKs) [14]. The results revealed that pictilisib treatment led to an increased accumulation of nuclear FOXO1 compared to vehicle-treated CRC cells, and the authors proposed FOXO1 as a putative co-target to rescue PI3Ki single-agent resistance in MCA therapy. In CRC, as well as for the majority of tumors, cancer stem cells (CSCs) are recognized as a primary contributor to drug resistance, tumor progression, and metastasis [15,16,17,18]. Several signaling pathways are implicated in maintaining cancer stemness; consequently, targeting these pathways emerges as a feasible strategy for eliminating CSCs and potentially tumor eradication [19]. Recently, some studies have shown that CD44, a cell surface glycoprotein, and its isoforms generated from alternative splicing involving standard and variant exons (CD44v) play a crucial role in the progression of CRC [20,21]. Notably, overexpression of CD44v6 is associated with an unfavorable prognosis in CRC patients, influencing adhesion, proliferation, stemness, invasiveness, and chemoresistance [22]. Accordingly, CD44v6 emerges as a promising target for both cancer diagnosis and therapy in CRC. Ejima et al. established a novel anti-CD44mAb, C44Mab-5 (IgG1, kappa), and C44Mab-46 (IgG1, kappa), and they evaluated their applicability through enzyme-linked immunosorbent assay, flow cytometry, western immunoblotting, and immunohistochemical analyses on several CRC cells [23]. Another widely recognized CSCs marker is ATP-binding cassette superfamily G member 2 (ABCG2), a multidrug transporter that mediates the translocation of diverse physiological and xenobiotic substrates across cellular membranes in an ATP-dependent manner [24]. The expression of the *ABCG2* gene has demonstrated negative prognostic implications in various malignancies, while in CRC, its prognostic significance remains undefined [25,26,27]. By analyzing publicly available datasets, Sałagacka-Kubiak et al. demonstrated that ABCG2 is downregulated in colon and rectum adenocarcinomas, exhibiting lower expression levels compared to both adjacent non-malignant tissues [28]. This deregulation is suggested to be associated with the methylation level of specific sites within the *ABCG2* gene and correlated with microsatellite instability (MSI), weight, and age, whilst in rectum adenocarcinoma patients, it was linked to tumor localization, population type, and age. Furthermore, an ABCG2-centered protein—protein interaction network, constructed using STRING, revealed that the associated proteins are involved in leukotriene, organic anion, xenobiotic transport, endodermal cell-fate specification, as well as histone methylation and ubiquitination. Therefore, the downregulation of ABCG2 may serve as a marker of the activity of specific signaling pathways or protein interactors crucial for colorectal carcinogenesis. Another protein engaged in CRC progression is the muscarinic acetylcholine receptor M3 (M3R) [29,30,31]. Analyzing 754 surgical CRC tissue samples, Lobbes et al. demonstrated that high expression of M3R correlated with enhanced survivability, particularly in cases with lower tumor grade and a non-mucinous subtype. This association was linked to a more favorable outcome compared to cases with low M3R expression, where survival significantly decreased and higher tumor grade and mucinous subtype were prevalent [32]. Genomic instability is a hallmark of CRC, and metastatic CRC (mCRC) characterized by deficient mismatch repair (dMMR) and MSI can effectively be treated using immune checkpoint inhibitors (ICI) such as pembrolizumab and nivolumab, approved by both the FDA and EMA [12,33,34]. Alternatively, combinations of programmed cell death protein-1 (PD-1) inhibitors with ipilimumab, an antibody targeting cytotoxic T-lymphocyte-associated protein 4 (CTLA-4), have also demonstrated efficacy in this context [35,36,37]. Krekeler et al. described the case of a 63-year-old male with microsatellite instability (MSI-H) and mCRC associated with Lynch syndrome [38]. The patient experienced rapid normalization of tumor markers and achieved a complete metabolic remission (CMR) that has persisted for ten months. This notable outcome was observed through a sequential ICI treatment approach involving the combination of nivolumab and ipilimumab, followed by nivolumab maintenance therapy after progression on single-agent PD-1 ICI therapy. This represents the first reported instance of sustained metabolic complete remission in an MSI-H mCRC patient who initially showed progression on single-agent anti-PD-1 therapy, suggesting that individuals with dMMR mCRC may have benefited from sequential immune checkpoint regimens, even exhibiting long-term responses.

In conclusion, personalized therapeutic regimens represent the cutting edge in CRC treatment. Strategies focused on targeting patient-specific markers have the potential to augment standard chemotherapy efficacy and mitigate tumor progression.
